# Morcellation Efficiency Across Prostate Size Categories in Holmium Laser Enucleation of the Prostate (HoLEP): A Complete-Case Analysis of 255 Consecutive Procedures at a UK Tertiary Centre

**DOI:** 10.7759/cureus.92533

**Published:** 2025-09-17

**Authors:** Momen Sid Ahmed, Nkwam Nkwam

**Affiliations:** 1 Department of Urology, King’s College Hospital NHS Foundation Trust, London, GBR

**Keywords:** benign prostatic hyperplasia, holep, mega-prostate, morcellation efficiency, operative time, prostate volume, surgical efficiency, trus

## Abstract

Background: Mechanical morcellation is integral to Holmium Laser Enucleation of the Prostate (HoLEP). Understanding how morcellation efficiency varies with gland size can inform list planning, device selection, and safety preparedness.

Objective: To quantify morcellation efficiency across preoperative prostate size categories and examine its association with gland volume.

Methods: We performed a retrospective, single-centre, complete-case analysis of 255 consecutive HoLEP procedures at a UK tertiary centre (January 2022-January 2025). Preoperative transrectal ultrasound (TRUS) prostate volume was categorised as <80 cc, 80-150 cc, and >150 cc. Morcellation efficiency (g/min) was calculated as enucleated tissue weight divided by morcellation time. Group differences were assessed nonparametrically with Kruskal-Wallis and Bonferroni-adjusted pairwise tests, and the relationship between volume and efficiency was evaluated with Spearman’s ρ.

Results: Size distribution was <80 cc 51 (20.0%), 80-150 cc 141 (55.3%), and >150 cc 63 (24.7%). Median morcellation efficiency decreased across categories: 9.02 (IQR 6.37-13.25) vs 8.71 (IQR 6.00-12.21) vs 7.00 g/min (IQR 4.25-9.57) (Kruskal-Wallis H=13.23, p=0.001); pairwise differences were significant for <80 vs >150 (p=0.005) and 80-150 vs >150 (p=0.003), but not <80 vs 80-150 (p>0.999). Efficiency inversely correlated with volume (ρ=−0.26, p<0.001).

Conclusions: Morcellation efficiency decreases as glands become very large (>150 cc); anticipating slower retrieval in mega-prostates can guide list composition, staffing, and morcellator/technique choice.

## Introduction

Holmium Laser Enucleation of the Prostate (HoLEP) is a guideline-endorsed, size-independent surgical option for bothersome lower urinary tract symptoms due to benign prostatic hyperplasia (BPH) [1]. The pairing of anatomical enucleation with mechanical morcellation established true size independence and broadened indications to very large glands [2,3]. Durable outcomes have been demonstrated over years of follow-up across the size spectrum [4]. Programs nevertheless face practical questions about retrieval throughput and safety when treating mega-prostates. Predictor analyses have linked tissue burden and consistency with morcellation time and rate, suggesting that very large glands may be logistically demanding even when overall outcomes remain excellent [5]. Treatment of prostates ≥200 cc has expanded, and reports emphasise feasibility alongside the operational challenges posed by the retrieval phase [6,7]. Comparative device studies and technique refinements indicate meaningful differences in morcellation performance that can be leveraged to optimise theatre flow [8-11]. Transrectal ultrasound (TRUS) is widely used to estimate preoperative prostate size for planning, and alternative retrieval strategies, such as pneumovesical extraction, can be considered when transurethral morcellation is constrained [12,13]. We evaluated morcellation efficiency across predefined TRUS size categories and assessed its association with preoperative volume in a consecutive, complete-case HoLEP cohort.

## Materials and methods

Study design

Single-centre retrospective analysis of consecutive HoLEP procedures using complete-case records for preoperative TRUS volume, enucleated weight, and morcellation time (N=255).

Study setting and duration

All eligible procedures performed during a continuous institutional audit window were screened. Consecutive cases meeting complete-case criteria were included without sampling. The study window spanned January 2022 through January 2025.

Study population

Adults undergoing HoLEP for symptomatic BPH were eligible. Procedures with missing preoperative TRUS volume, morcellation time, or enucleated weight were excluded.

Data collection

Data were abstracted from operative notes, anaesthetic charts, pathology records, and clinic ultrasound reports. Core variables included preoperative TRUS volume (cc), enucleated tissue weight (g), and morcellation time (min). A de-identified dataset was assembled and checked for outliers and internal consistency before analysis.

Definitions and derived measures

The primary endpoint was morcellation efficiency (ME), defined as enucleated tissue weight divided by morcellation time $\mathrm{ME}=\frac{W_{enuc}(g)}{T_{morc}(min)}$. Prespecified prostate size categories were <80 cc, 80-150 cc, and >150 cc. All measures were abstracted from routine clinical documentation exactly as recorded, and complete-case criteria were applied prior to analysis.

Statistical analysis

Continuous variables are presented as mean ± SD and median (IQR); categorical variables as n (%). Between-group comparisons of morcellation efficiency used Kruskal-Wallis tests with Bonferroni-adjusted Mann-Whitney U tests (three contrasts). The association between preoperative volume and morcellation efficiency was assessed using Spearman’s ρ. Two-sided α=0.05. No external sources are cited in this section.

## Results

Cohort and descriptive characteristics

We analysed 255 (100.0%) complete cases. Preoperative TRUS volume categories were <80 cc 51 (20.0%), 80-150 cc 141 (55.3%), and >150 cc 63 (24.7%). Group-wise summaries (volume, enucleated weight, enucleation/morcellation times, and efficiencies) are presented in Table 1.

**Table 1:** Morcellation metrics by prostate size group Morcellation efficiency = enucleated weight ÷ morcellation time (g/min); enucleation efficiency = enucleated weight ÷ enucleation time (g/min).

Size group	n (all)	n with valid efficiency	Prostate volume, mean ± SD (cc)	Enucleated weight, mean ± SD (g)	Enucleation time, mean ± SD (min)	Morcellation time, mean ± SD (min)	Enucleation efficiency, mean ± SD (g/min)	Morcellation efficiency, mean ± SD (g/min)
<80 cc	51	50	61.8 ± 12.5	44.3 ± 20.5	31.1 ± 13.6	5.3 ± 3.6	1.68 ± 1.00	10.07 ± 4.90
80–150 cc	141	141	115.6 ± 16.8	81.1 ± 25.6	42.7 ± 20.3	11.4 ± 8.0	2.19 ± 0.98	9.32 ± 4.48
>150 cc	63	63	200.4 ± 54.5	140.4 ± 49.8	58.3 ± 24.1	25.6 ± 18.4	2.62 ± 0.93	7.53 ± 5.16

Morcellation efficiency by size

**Figure 1:**
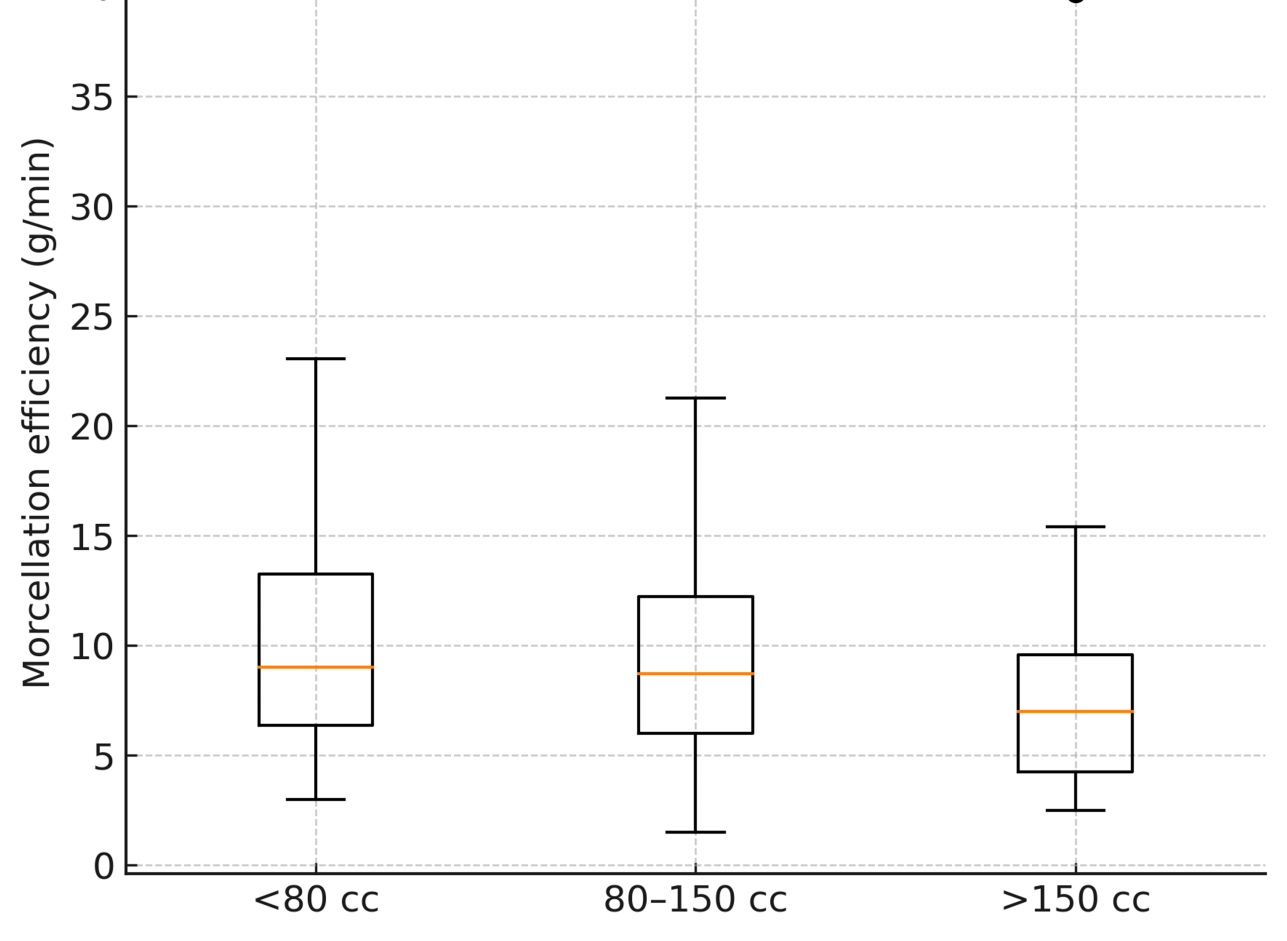
Morcellation efficiency by prostate size group Violin/box overlay of morcellation efficiency (g/min) across <80 cc, 80–150 cc, and >150 cc groups.

Median morcellation efficiency decreased across size categories: 9.02 (IQR 6.37-13.25) for <80 cc, 8.71 (IQR 6.00-12.21) for 80-150 cc, and 7.00 (IQR 4.25-9.57) for >150 cc (Kruskal-Wallis H=13.23, p=0.001). Bonferroni-adjusted pairwise tests were significant for <80 vs >150 (p=0.005) and 80-150 vs >150 (p=0.003), but not <80 vs 80-150 (p>0.999). The distribution across categories is displayed in Figure 1.

Relationship between volume and morcellation efficiency

**Figure 2:**
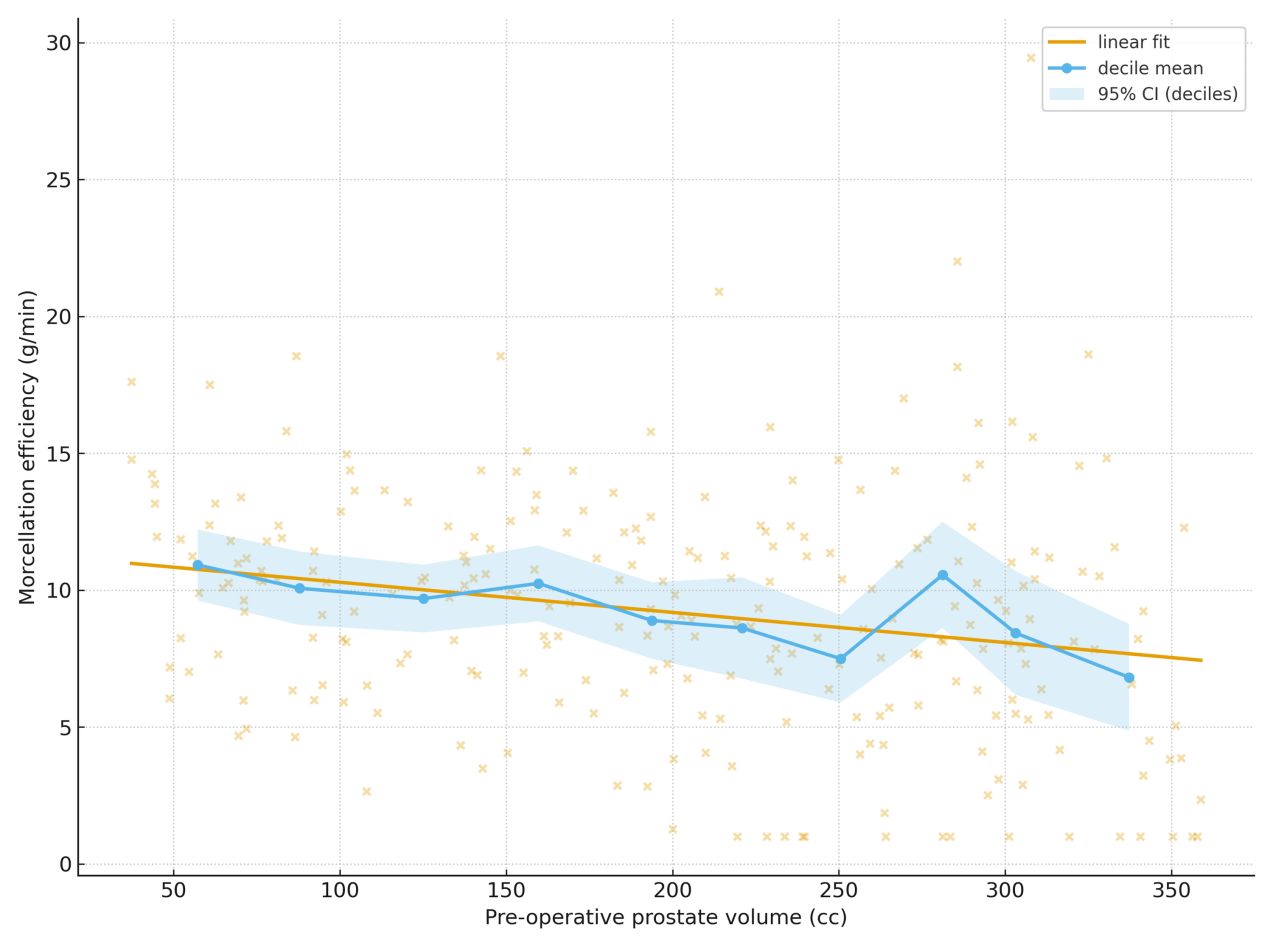
Morcellation efficiency versus preoperative prostate volume Scatter of morcellation efficiency (g/min) against pre-operative prostate volume (cc) with a linear fit and decile means (95% CI).

Morcellation efficiency inversely correlated with preoperative TRUS volume (Spearman ρ = −0.26, p<0.001). The continuous relationship is visualised in Figure 2.

## Discussion

Principal findings and clinical meaning

In this consecutive complete-case cohort, morcellation efficiency declined as gland size increased, with the largest decrement in >150 cc prostates. These findings fit with the concept that larger, often more fibrotic adenomas slow retrieval despite HoLEP’s size-independent efficacy [1-4].

Alignment with prior work

Predictor analyses have reported associations between tissue burden/consistency and slower morcellation, which is in line with our inverse correlation between volume and efficiency [5]. As programs treat more mega-prostates, published series underline feasibility while acknowledging the logistical demands of retrieval; our size-stratified analysis quantifies this effect and highlights the threshold at which it becomes most apparent [6,7].

Devices and techniques

Randomised and comparative studies indicate measurable performance differences between morcellators; centres may achieve clinically meaningful gains in large-gland lists by selecting higher-throughput systems [8-10]. Technique refinements, for example, “inverse” morcellation, have been associated with efficiency and safety benefits and may attenuate size-related slowdowns in experienced hands [11].

Context within size-independent outcomes

HoLEP delivers durable outcomes across sizes, including very large prostates, and remains an alternative to open prostatectomy and resection in large glands; our data support optimising retrieval logistics rather than altering indications [14-16]. Learning-curve studies emphasise that morcellation proficiency is achieved relatively early, potentially containing variability in mid-sized glands [17,18]. Analyses across gland weights further contextualise throughput, suggesting a trade-off whereby enucleation efficiency may rise while morcellation efficiency falls as weight increases-our results align with the morcellation component of that trade-off [19]. Technical updates continue to detail steps that improve safety and flow during enucleation and retrieval [20].

Strengths and limitations

Strengths include complete-case inclusion, clinically meaningful size strata that isolate mega-prostates, and nonparametric statistics suited to skewed distributions. Limitations include single-centre design and no stratification by morcellator model; however, the magnitude and direction of effects are consistent with randomised and comparative device studies and established predictors work [5,8-10].

## Conclusions

Morcellation efficiency decreases as preoperative gland size increases, with the most pronounced slowing above 150 cc. In practical terms, theatre lists that include mega-prostates should budget additional retrieval time and consider higher-throughput morcellators or alternative strategies to maintain overall flow and safety. These data also support proactive staffing and equipment planning for large-gland sessions without changing HoLEP candidacy. Future audit cycles will examine device-level differences and workflow adjustments to mitigate size-related slowdowns.
